# An oligonucleotide synthesizer based on a microreactor chip and an inkjet printer

**DOI:** 10.1038/s41598-019-41519-0

**Published:** 2019-03-25

**Authors:** Hui Li, Ye Huang, Zewen Wei, Wei Wang, Zhenjun Yang, Zicai Liang, Zhihong Li

**Affiliations:** 1 Institute of Microelectronics, Peking University, Beijing, China; 2 State Key Laboratory of Natural and Biomimetic Drugs, School of Pharmaceutical Sciences, Peking University, Beijing, China; 3 National Center for Nanoscience and Technology, Beijing, China; 4 Institute of Molecular Medicine, Peking University, Beijing, China; 5 Department of Biomedical Engineering, Pennsylvania State University, University Park, PA United States; 6 Department of New Drug Research and Development, Institute of Materia Medica, Peking Union Medical College & Chinese Academy of Medical Sciences, Beijing, China

## Abstract

Synthetic oligonucleotides (oligos) are important tools in the fields of molecular biology and genetic engineering. For applications requiring a large number of oligos with high concentration, it is critical to perform high throughput oligo synthesis and achieve high yield of each oligo. This study reports a microreactor chip for oligo synthesis. By incorporating silica beads in the microreactors, the surface area of the solid substrate for oligo synthesis increases significantly in each microreactor. These beads are fixed in the microreactors to withstand the flushing step in oligo synthesis. Compared to conventional synthesis methods, this design is able to avoid protocols to hold the beads and integrate more microreactors on a chip. An inkjet printer is utilized to deliver chemical reagents in the microreactors. To evaluate the feasibility of oligo synthesis using this proof-of-concept synthesizer, an oligo with six nucleotide units is successfully synthesized.

## Introduction

Synthetic oligonucleotide (oligo) serves as a unique tool to explore molecular biology, protein engineering, and genetic diagnosis^1–3^. The four-step, solid-phase phosphoramidite cycle is the standard method for current oligo synthesis^4,5^. With the initial phosphoramidite nucleotide fixed on a solid substrate, the target oligo is synthesized by coupling new nucleotides. To achieve high product yield, controlled pore glass (CPG) is widely employed due to the significant enhancement of surface area. As the oligo synthesis is a cyclic process, the CPG is maintained in physical separated columns while the chemical waste between adjacent reactions is flushed away. The nature of this process minimizes the cross contamination between columns and leads to high quality products. This column-based process can typically automate 96–1536 oligos synthesis in parallel^6^.

Microfluidics provides opportunities to achieve high throughput oligo synthesis at low cost^6–9^. Microarray-based oligo synthesis is well known for the extremely high throughput that can be as high as tens of thousands of oligos per chip^10^. In particular, oligo synthesis is performed at specific locations on the surface using light-activated reactions, inkjet printing technology, or electrochemical reactions^11–16^. The concentration of individual oligos is relatively low (i.e., at the femtomolar level) and an amplification step (e.g., polymerase chain reaction (PCR)) is required for further applications^17–21^. To enhance the synthetic amount of each oligo, engineering designs, including paper-based devices, engineering filters, microfluidic valves, and optical tweezers, have been developed to generate *in situ* micro-columns to increase the surface area for the oligo synthesis^22–27^. However, these techniques do not fully address the challenge for simply and practically oligo synthesis.

In this study, we develop a microreactor chip for oligo synthesis that integrates fixed silica beads in the microreactors. The microreactors are fabricated on a silicon wafer using an anisotropic etching process. Silica beads are self-assembled in the microreactors to increase the surface area. The surface area of each microreactor with beads is 71 times more than an empty microreactor. As oligo synthesis occurs on the surface of the beads, the surface area is critical to the product yield. These beads are sintered and fixed in the microreactors, creating high mechanical strength bonds among the beads and between the beads and the microreactor walls. This design is able to retain the beads during flushing processes between reactions in oligo synthesis. An inkjet printer is utilized to deliver chemical reagents in the microreactors. As a proof-of-concept, an oligo with six phosphoramidite monomers is successfully synthesized using the proposed platform.

## Results

### Design of the oligo synthesizer

**Figure 1:**
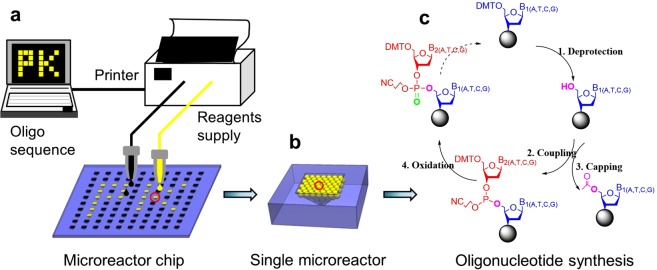
The schematic view of the oligonucleotide synthesis on our platform. (**a**) The synthesis processes involve designing target sequence, delivering chemical reagents through the inkjet printer, and oligonucleotide synthesis in the microreactor chip. (**b**) The single microreactor is filled with silica beads which enhance the surface area for the following synthesis. The beads are inherently fixed in the microreactor using sintering process. (**c**) The oligonucleotide synthesis on the silica beads follows the four-step with phosphoramidite strategy: deprotection, coupling, capping and oxidation.

The oligo synthesizer has been developed using a microreactor chip and an inkjet printer (Fig. 1a). The chip integrates an array of microreactors which are used to synthesize various oligos in parallel. A commercial inkjet printer is applied to deliver chemical reagents into the microreactors according to the oligo sequence design. To enhance the product yield in each microreactor, silica beads are employed to increase the surface area (Fig. 1b). The beads are physically immobilized in the microreactor using a sintering process and can inherently withstand the harsh washing step during oligo synthesis. Synthesis on the beads follows the four-step, solid-phase phosphoramidite cycle, including deprotection, coupling, capping, and oxidation (Fig. 1c). The chemical waste is removed through the distributed microchannels networks among the beads after each reaction.

### Microreactor chip

**Figure 2:**
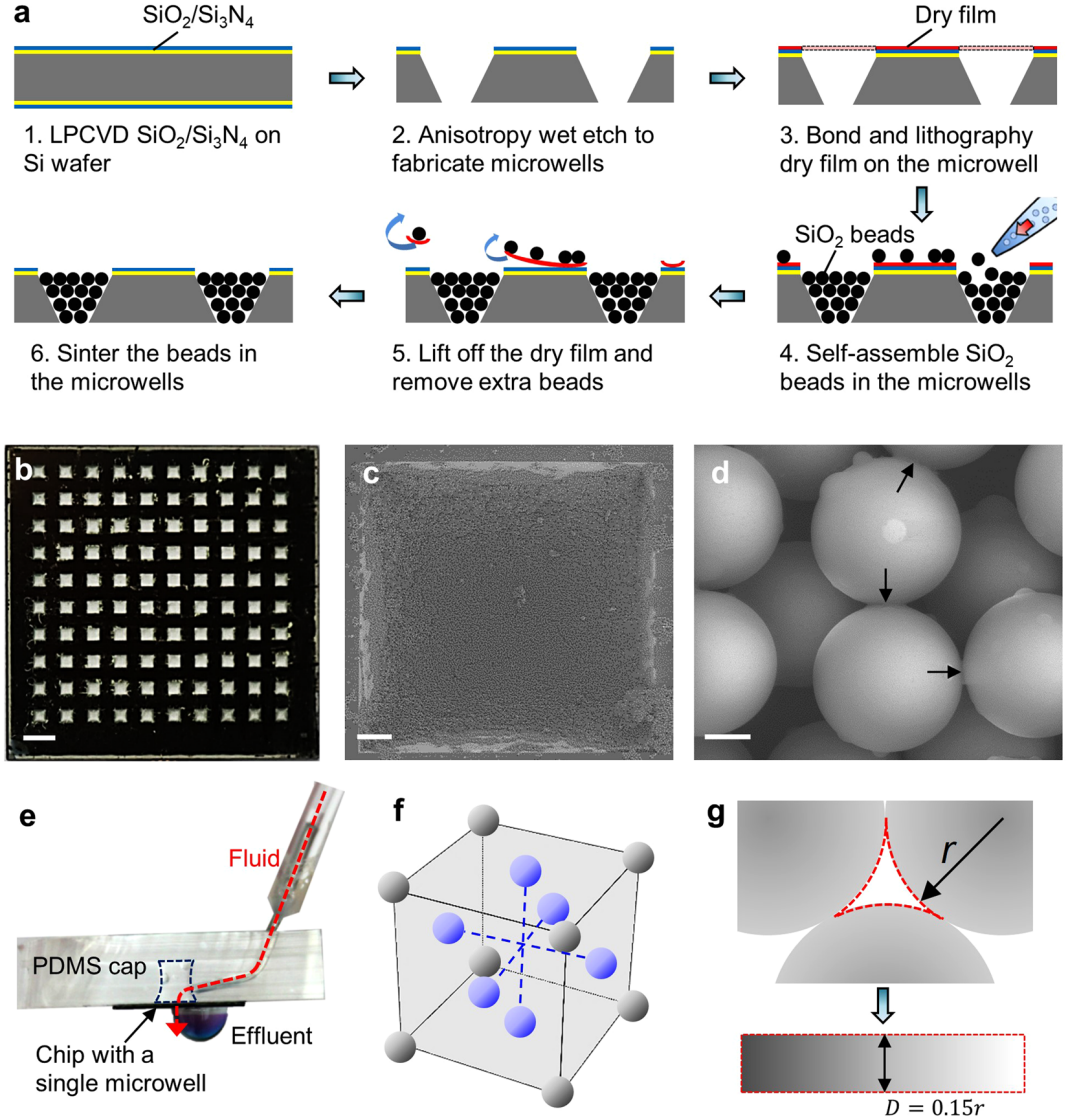
The microreactor array chip fabrication and characterization. (**a**) The fabrication processes to prepare the microreactor array chip: microwells were fabricated on substrate (1–2); a layer of dry film was bonded and patterned on the microwells (3); silica beads were self-assembled and fixed within the microwells and extra beads among microwells were removed with the dry film (4–6). (**b**) The photograph of a microreactor array chip. (**c**) SEM image of a single microreactor. (**d**) SEM image showing the physical bonding among silica beads. Scale bars in (**b**), (**c**), and (**d**) are 2 mm, 100 μm, and 2 μm, respectively. (**e**)The setup to test the mechanical strength of the beads in the microreactor. (**f**) The physical model of the packed beads and (**g**) the distributed microchannel network among the beads.

**Figure 3:**
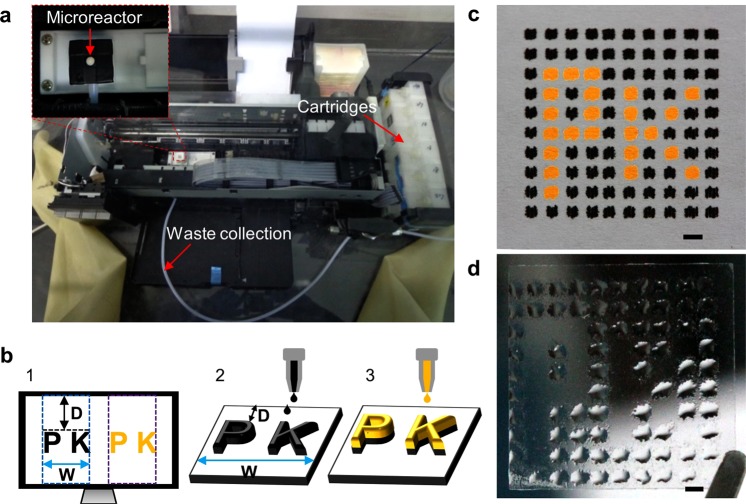
The reagent delivery system utilizing a commercial inkjet printer. (**a**) The photograph of the reagent delivery system. The cartridges were loaded with chemical reagents which can be delivered to the microreactor chip under the printer heads. A vacuum waste collection was employed to collect waste reagents in the microreactor. The system was stored in a nitrogen dry glove box. (**b**) The projection between the target pattern in Microsoft PowerPoint (1) and the printed reagents on an immobilized substrate (2–3). The position of the reagents on the substrate can be tuned and different reagents can be printed to the same position. (**c**) Pigment ink pattern on an immobilized paper after 40 repeat printing cycles on each point. (**d**) Acetonitrile pattern on an immobilized cover slide after 40 repeat printing cycles on each point. Scale bars in (**c**) and (**d**), 2 mm.

**Figure 4:**
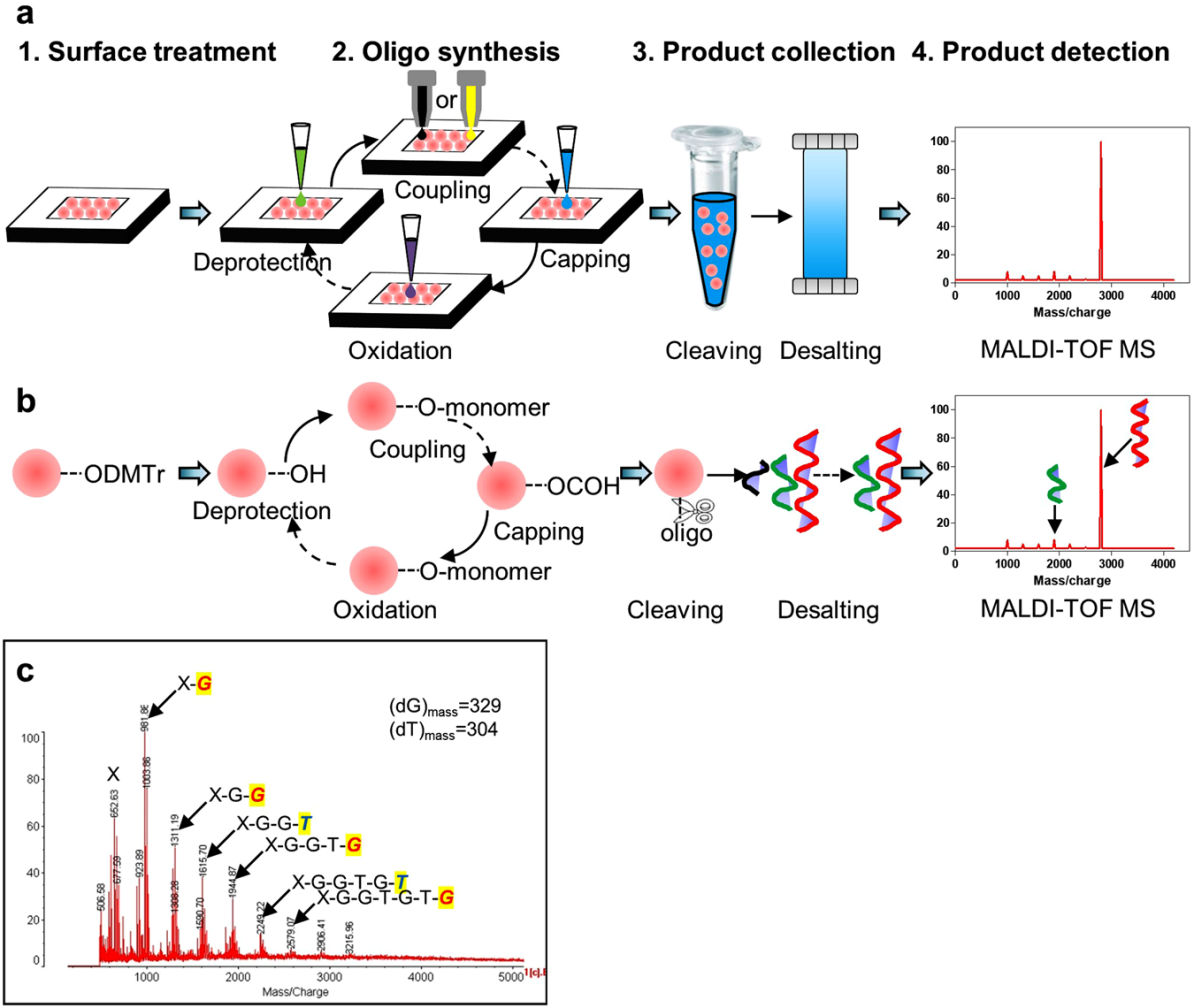
Oligonucleotide synthesis using the microreactor chip and the printer-based reagent delivery system. (**a**) The oligonucleotide synthesis includes surface treatment of the beads in the microreactors (1), the 4-step oligo synthesis (2), product collection (3), and product detection (4). (**b**) The respective chemical reactions on a single bead corresponding to (**a**). (**c**) Mass spectrometry of the synthesized oligo on the platform. There are six oligos with 1-6 nucleotide units and the sequence is determined from the mass difference of two adjacent peaks, respectively.

We fabricated the microreactor chip by assembling silica beads in the microreactor (Fig. 2a). First, silicon dioxide (100 nm) and silicon nitride (100 nm) were deposited on a silicon wafer using low pressure chemical vapor deposition (LPCVD) (Fig. 2a-1). Second, the silicon dioxide and silicon nitride were patterned using reactive ion etching (RIE). The aperture size is 700 μm × 700 μm. Third, the wedge-shaped microreactors were formed by KOH etching for 420 min. As the angle between the sidewall and the wafer surface is 54.7°, the bottom aperture size of the microreactor is 134 μm × 134 μm. The back silicon oxide and silicon nitride were then removed using RIE (Fig. 2a-2). Next, a dry film was bonded with the substrate at 110 °C and patterned using a standard lithography process. This process prevented photoresist buildup in the microreactors, which would occur in the conventional spin-coating process (Fig. 2a-3). Silica beads were pipetted and self-assembled in the microreactors (Fig. 2a-4)^28–30^. The dry film was lift-off from the substrate by applying acetone for 1 min to curve up the film. The extra beads between adjacent microreactors were removed due to lift-off of the bottom dry film (Fig. 2a-5). This process avoids microchannel generation between microreactors and prevents potential cross-contaminations during the synthesis process. Finally, the beads were fixed within the microreactor after sintering at 1050 °C for 12 hours (Fig. 2a-6)^31,32^. The silica beads can maintain the spherical shape and start to melt only on the surface. The melting material bonds the beads together.

The microreactor chip was characterized. The chip integrates 10 × 10 individual microreactors in a 20 mm × 20 mm area (Fig. 2b). Each microreactor is 700 μm × 700 μm and the interval between the microreactors is 1000 μm. The beads were closely packed in the individual microreactor (Fig. 2c). Compared with a microreactor that consists of 4 sidewalls (${S}_{0}=0.008\,c{m}^{2}$), the surface area of this bead-packed microreactor is increased by 71 times (${S}_{1}=0.57\,c{m}^{2}$). This is due to the large surface area of the beads, which can be evaluated by the number of the beads (Eq. 1) and the surface area of a single bead (Eq. 2). Taking advantage of this design, the oligo yield can be potentially increased by 71 times in each microreactor. To hold the beads in the washing step of oligo synthesis, the beads were sintered and immobilized in the microreactor. The physical bonding among the beads indicates that the beads are physically separated and the surface melting bonds them together (Fig. 2d).

1 $${{\rm{N}}}_{beads}=\frac{Equavilent\,chamber\,volume}{Volume\,of\,a\,bead}=\frac{\frac{1}{3}({S}_{1}{h}_{1}-{S}_{2}{h}_{2})\times 0.74}{\frac{4}{3}\pi {r}^{3}}=4.5\times {10}^{5}$$

2 $${{\rm{S}}}_{bead}=4{\rm{\pi }}{r}^{2}=124.6\,\mu {m}^{2}$$

The mechanical strength of the porous structure was studied with high velocity fluid (Fig. 2e). A device with a single microreactor was bonded with a layer of PDMS after 5 min oxygen plasma treatment. A tube was connected to the microreactor through the PDMS layer. Distilled water was applied to flush the beads in the microreactor from a syringe pump. The loading rate was 0.2 mL/s and the loading time was 30 s. There was no visible damage to the packed beads after this process (data not shown). To study the correlation between this flush process and the washing step in the oligo synthesis, the distributed microchannels through the packed beads were calculated. The beads were packed in face-centered cubic structure in the microreactor (Fig. 2f)^33^. The channel network among the beads was modelled as an equivalent straight channel with feature diameter of 15% of the bead diameter (Fig. 2g)^30^. The pressure difference across the microreactor was 191 kPa, which was determined by the fluid rate and channel size using Poiseuille’s law (Eq. 3). This pressure difference was higher than the pressure generated from the vacuum pump (Rotehoo, max pressure = 88 kPa) in the washing step. This correlation verified that the beads are able to withstand the washing step in the oligo synthesis.

3 $${\rm{\Delta }}P=\frac{8\eta L\cdot Q}{\pi {r}^{4}}=1.91\times {10}^{5}\,{\rm{Pa}}$$

Here, ${\rm{\Delta }}P$, $\eta$, *L*, *Q*, and *r* represent the pressure difference, fluid viscosity, length of the channels, flow rate, and radius of the channels, respectively.

### Inkjet printer based reagent delivery system

We developed an inkjet printer-based reagent delivery system to automatically and accurately deliver chemical reagents into the microreactors (Fig. 3a). This system included three parts: (a) two cartridges loaded with reagents; (b) the microreactor chip on the Teflon platform under the nozzles (inset); (c) a waste collection system. We first characterized the relationship between the input patterns on Microsoft PowerPoint and the output reagents on substrate (Fig. 3b). The whole patterns are calculated and divided into columns from left to right prior to the printing (Fig. 3b-1). The width of each column equals the width of the nozzle array (W) and the first column aligns with the first left pattern. The blanks between adjacent columns are excluded (e.g., the gap between the blue and purple columns). Each column of the patterns is printed from top to bottom onto the substrate and the y-axis of the droplet is determined from the y-axis of the patterns (D) (Fig. 3b-2). Therefore, the location of the reagent can be tuned by adjusting the input patterns. With proper pattern design (e.g., repeat columns and adjust the pattern color), reagents can be selectively printed to the precise location on the substrate (Fig. 3b-3). This ensures sufficient reagents at each location and facilitates reagent mixing at the nanoliter level.

The feasibility of this reagent delivery system was validated by printing pigment ink and acetonitrile patterns. Pigment inks were loaded in corresponding cartridges and printed on a paper fixed on the Teflon platform. The pattern was designed to be identical to the microreactor chip. The size of each spot is 700 μm × 700 μm, and the interval between adjacent spots is 1000 μm. To project the input patterns onto the fixed paper, the location of the Teflon platform was carefully adjusted according to the working principle (Fig. 3b) and fixed in the following experiments. To mimic the printing in oligo synthesis, which requires 21 nL reagent to fill the single microreactor, we evaluated the liquid volume in an individual printing and performed repetition printing on each spot. There was around 9.5 nL reagent printed on each spot in a single printing process, which was calculated based on the single droplet ejected from a nozzle (1.5 pL), the resolution of the printer (5760 × 1440 dpi), and the spot size (700 μm × 700 μm). To verify the alignment in repetition printing, the pattern with two pigment inks was obtained after 40 repeating printings on each spot (Fig. 3c). The single spot was (1227 ± 157) μm × (1051 ± 131) μm and the interval was 779 ± 126 μm. The data represent mean ± SD (n = 12). To note, there was no overlap between spots and the minor misalignment can be reduced by changing the pattern design. With the same pattern design and printer setting, acetonitrile was loaded in the black cartridge (the other cartridges were blank) and printed on a cover slide (Fig. 3d). The single spot was (1671 ± 204) μm × (1510 ± 125) μm and the interval was 539 ± 140 μm without mixing. The data represent mean ± SD (n = 12). Compared with the patterns on paper, acetonitrile had a tendency to spread on the cover slide due to surface tension.

### Oligo synthesis on the chip

To demonstrate the prototype application, oligo synthesis was implemented on the chip with a single microreactor. The workflow and the corresponding chemical reactions are illustrated (Fig. 4a,b). The surface treatment was applied to initiate active chemical bonding for the following oligo synthesis (Fig. 4a,b-1). The detailed information can be found in the materials and methods section. The four-step, solid-phase phosphoramidite cycle was subsequently performed on the chip, including deprotection, coupling, capping, and oxidation (Fig. 4a-2). The chemical reactions are explained in Fig. 4b-2 and the materials and methods section. With different monomers coupled on the oligo, the synthesis was repetitively performed to achieve a target sequence. The synthesized oligo was cleaved from the beads and purified by desalting (Fig. 4a,b-3 and the materials and methods section). Finally, the product was analyzed using matrix assisted laser desorption ionization-time of flight mass spectrometry (MALDI-TOF MS, Fig. 4a,b-4).

We successfully synthesized an oligo with six phosphoramidite monomers on-chip following the above workflow (Fig. 4c). In this case, each mass peak represents a product and the difference between adjacent peaks denotes the mass difference of corresponding products. This mass difference indicates the monomer with the same molar mass. Therefore, the oligo sequence was determined as “X-GGTGTG” (Fig. 4c). This sequence was in agreement with our design.

## Discussion

In this study, we developed a microreactor chip for oligo synthesis. This chip integrates fixed silica beads in the microreactors. Owing to the high surface to volume ratio of the beads, the surface area in each microreactor increases by 71 times compared to microreactors without beads. The increased surface area significantly improves the product yield. The beads were fixed within the microreactors and can resist flushing processes in oligo synthesis. To evaluate the feasibility of oligo synthesis using this microreactor chip, an oligo with six nucleotide units was successfully synthesized.

As we demonstrated the methodology of building up the oligo synthesizer and validated the feasibility of oligo synthesis using the proposed synthesizer, the synthesis efficiency should be further improved. The synthesis efficiency in each step could be evaluated by the relative amount of the terminated oligos (Table S1). For instance, the synthesis efficiency of the first monomer could be calculated by the relative amount of oligos from 1 monomer to 6 monomers divided by the relative amount of oligos from the initial base to 6 monomers. Compared to commercialized systems, this result indicated a relatively low synthesis efficiency. There are several possibilities to improve the efficiency. First, adequate washing may help to reduce crosstalk between adjacent reactions that caused by the chemicals in the dead ends of the distributed microchannels. Then, the synthesis efficiency can be enhanced by minimizing moisture and oxygen in the environment. The relationship between the synthesis efficiency and the sintering process in the fabrication should be studied and characterized. With high synthesis efficiency, longer oligos will be synthesized, the product amount can be determined in the beads filled microreactors and compared with the blank reactors and commercial systems, and the synthesis throughput should be scaled up.

There are other approaches to improve system performance. With different types of beads with a lower melting temperature, the sintering process can be optimized to a lower temperature and less process time. The synthesis processes can be automated with commercial reagent delivery systems, which would also reduce reagent consumption and synthesis cost.

## Materials and Methods

### Microreactor chip

The silicon wafer is in (100) crystal direction and 400 μm in thickness. The dry film (Dupont Riston FX530) was obtained from Dupont. The thickness of the dry film is 30 μm. The silica beads were purchased from Unisize Technology (Changzhou) Co., Ltd. The diameter of the beads is 6.3 μm. To avoid cracks during the following sintering process, the beads were pretreated at 600 °C for 4 hours to remove surface physisorbed water before assembly in the microreactor^31,34,35^. Polydimethylsiloxane (PDMS, Sylgard 184) was obtained from Dow Corning.

### Inkjet printer-based reagents delivery system

The inkjet printer (Epson, L800) was purchased from Epson. This printer has six sets of identical piezoelectric printer heads that are able to independently deliver reagents through corresponding cartridges. The compatibility of the chemical reagents and the printer was examined by successfully printing acetonitrile (Sigma) stored in the black cartridge.

To achieve alignment between the printed reagents and the substrate (e.g., the microreactor chip, paper, and glass cover slide), the substrate was immobilized on a Teflon frame. By tuning the height of the Teflon frame, the gap between the substrate and the printer head was reduced to approximately 2 mm. This reduces lateral movement of the printing droplet and facilitates accurate printing. To mimic the washing step in oligo synthesis, the reagents in the microreactor can be collected from the backside of the chip that was placed on a Teflon frame and connected with a vacuum pump.

### Surface treatment of the beads

A chemical surface treatment was applied to initiate chemical bonding on the sintered beads for oligo synthesis. The detailed surface treatment is illustrated in the following reactions (Fig. S1). First, the beads were hydroxylated in Piranha solution, which is a mixture of sulfuric acid (H_2_SO_4_) and hydrogen peroxide (H_2_O_2_) at a ratio of 3:1, at 100 °C for 1 hr. Then, the beads were subsequently aminated in a solution of (3-Aminopropyl)triethoxysilane (ATPES) and toluene at a ratio of 1:10 at 50 °C for 16 hr. Next, a spacer was bonded on the amino group. The spacer was prepared with 1-hydroxybenzotriazole (HOBT), 2-(1H-Benzotriazole-1-yl)-1,1,3,3-tetramethyluronium hexafluorophosphate (HBTU), N,N-Diisopropylethylamine (DIPEA) in N,N-Dimethylformamide (DMF). The final concentration of the above four chemicals were 30 mM. This reagent was activated at 28 °C for 5 min before use. The beads were treated in this reagent at 28 °C for 3 hr. Finally, a linker was bonded to facilitate the product collection after the synthesis process. The linker was thoroughly mixed with 4-dimethylaminopyridine (DMAP), N,N-Dimethylformamide (DMF), and dichloromethane (DCM). The amount was 0.15 mmol, 0.03 mmol, 2.25 mL, and 0.75 mL, respectively. This reagent was mixed with dicyclohexylcarbodiimide (DCC, 1 M in DCM) at a ratio of 10:1 (v/v). The beads were treated in this reagent at 37 °C for 24 hr.

### Oligo synthesis on chip

The chemicals for oligo synthesis involve deblocking, capping, and oxidation reagents, and phosphoramidite monomers. The deblocking reagent was trichloroacetic acid (TCA) in dichloromethane (DCM) (3%, w/v), capping reagents contained capping A and capping B (1:1, v/v), phosphoramidite monomers were phosphoramidite monomers with tetrazole (1:1, v/v), and oxidation reagent was iodine (0.015 M) in water/pyridine/THF (2/20/78, v/v/v). The capping A was acetic anhydride/pyridine/THF (1/1/8, v/v/v)) and capping B was N-methyl imidazole in acetonitrile (17.6%, w/v). The washing reagent was acetonitrile. The reagents to cleave product from the silica beads was aqueous ammonium hydroxide and aqueous methylamine (1:1, v/v).

Oligo synthesis was performed with this microreactor chip. After the surface treatment, 10 μL deprotecting reagent was pipetted to the single microreactor to remove the dimethyl terephthalate (DMT) and initiate active hydroxyl bonds on the silica beads. This reaction was set to 1 min. Target phosphoramidite monomer was printed into the microreactor to couple on the active hydroxyl bonds. In this case, the dG and dT were delivered from the black and yellow cartridges. The target was printed 40 times. This process was performed by printing 5 times of a Microsoft PowerPoint slide that consisted of 8 same patterns (i.e., each pattern consisted of a 700 μm × 700 μm spot and a negligible alignment mark). This process took <15 s. To address the potential nozzle clogging issue, the printer heads were cleaned using the printer control software at the beginning of the reagent delivery process. The reagent occupied the distribution microchannels among the beads and the chemical reaction was accomplished on the beads surface. The reaction was 2 min after the printing. There was no significant evaporation observed in this process. It might be attributed to the short time and the capability of reagent preservation in the microchannels. The capping process was subsequently performed by pipetting 10 μL capping reagents in the microreactor to esterify failure sequences and prevent them for the following reactions. This step was set to 1 min. The phosphite internucleotide linkage was oxidized to the phosphotriester by pipetting 10 μL oxidation reagent to the microreactor. This step was set to 1 min. Flushing process was repeated 3 times between adjacent reactions using 100 μL acetonitrile. Repetitions of this four-step process with different phosphoramidite monomers produced the oligonucleotide with specific sequence. All the synthesis processes were performed in a nitrogen dry glove box. The oligo product was cleaved from the beads by immersing the chip in cleaving reagents at 60 °C for 2 hr. The product was purified with desalting and characterized with MALDI-TOF MS.

## Supplementary information

Supplementary information- An oligonucleotide synthesizer based on a microreactor chip and an inkjet printer

## Data Availability

Contact Z.H.L. for additional information about the proposed system.
