# Supplementary material for: An oligonucleotide synthesizer based on a microreactor chip and an inkjet printer
Source: Sci Rep. 2019 Mar 25;9:5058. doi: 10.1038/s41598-019-41519-0 (PMC6434144; doi:10.1038/s41598-019-41519-0)
Supplement: Supplementary file 1 — Supplementary information- An oligonucleotide synthesizer based on a microreactor chip and an inkjet printer [file 41598_2019_41519_MOESM1_ESM.pdf]

Supplementary information

An oligonucleotide synthesizer based on a microreactor chip and an inkjet printer

Hui Li[1,5†], Ye Huang[2,6†], Zewen Wei[3], Wei Wang[1], Zhenjun Yang[2*], Zicai Liang[4*] and Zhihong Li[1*]

1 Institute of Microelectronics, Peking University, Beijing, China

2 State Key Laboratory of Natural and Biomimetic Drugs, School of Pharmaceutical Sciences, Peking University, Beijing, China

3 National Center for Nanoscience and Technology, Beijing, China

4 Institute of Molecular Medicine, Peking University, Beijing, China

5 Department of Biomedical Engineering, Pennsylvania State University, PA, USA

6 Department of New Drug Research and Development, Institute of Materia Medica, Peking Union Medical College & Chinese Academy of Medical Sciences, Beijing, China.

†Those authors contributed equally to this paper.

*Corresponding Author: Zhihong Li (Email: zhhli@pku.edu.cn; Tel: +86-10-62766581), Zhenjun Yang (Email: yangzj@bjmu.edu.cn; Tel: 86-10-82802503), or Zicai Liang (Email: Liangz@pku.edu.cn; Tel: 86-10-62769862).

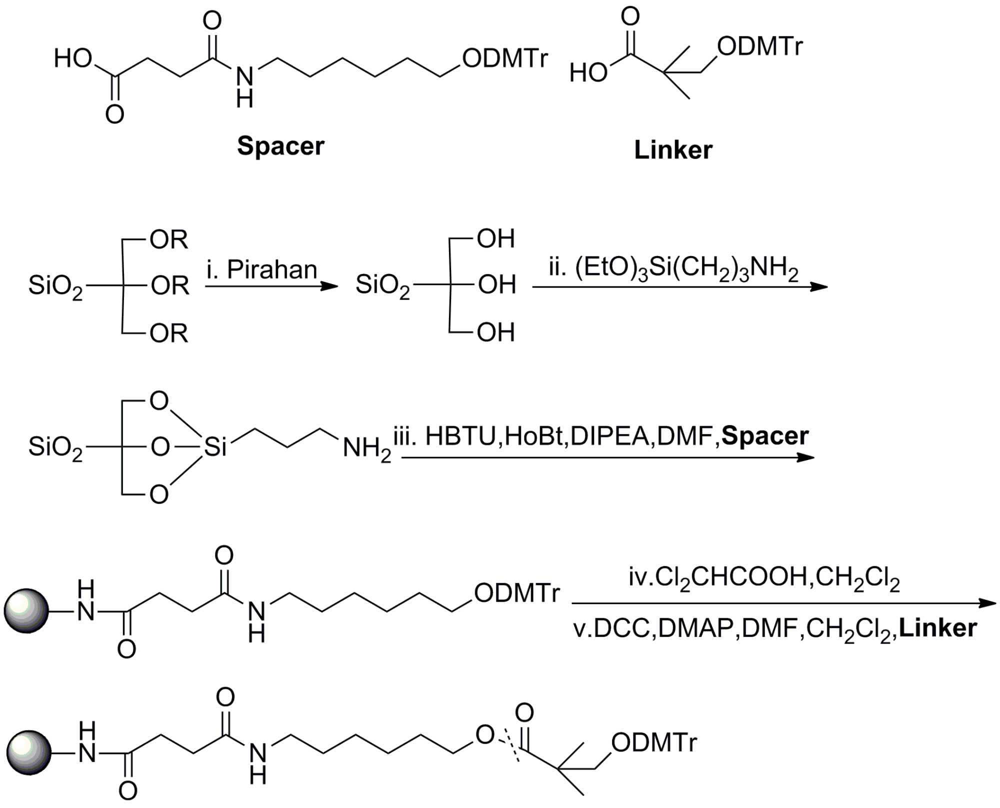

Fig. S1 Surface treatment of the sintered beads for oligo synthesis.

Table S1. The relative oligo amount and synthesis efficiency in each step

Oligos	Relative amount	Synthesis efficiency
Initial	65	NA
1 monomer	100	79%
2 monomers	52	59%
3 monomers	39	63%
4 monomers	29	56%
5 monomers	14	43%
6 monomers	7.5	35%
